# Retraction: Functional Conservation of the *Drosophila gooseberry* Gene and Its Evolutionary Alleles

**DOI:** 10.1371/annotation/3ca44f3b-db28-4d64-88e4-1a536aa95174

**Published:** 2012-06-29

**Authors:** Wei Liu, Lei Xue

Following the publication of this article, a number of concerns were brought to the attention of the Editors of PLoS ONE in relation to this research. In particular, the reliability of the data reported in Tables 1 and 2 has been questioned. The authors have indicated that they do not have access to the raw data on which the results are based, as a result, the authors retract the article given that it is currently not possible to verify the validity of the results.

Details on the specific concerns raised on the article are outlined below:

- The contents of the manuscript have been taken almost entirely from one of the chapters of the first author’s PhD thesis, which was carried out under Dr Markus Noll’s supervision at the University of Zürich. However, the authors did not discuss the contents of the manuscript with him and he was not included as an author.

- The references to Markus and Hans Noll under the Acknowledgements are inadequate as they were not shown a copy of the manuscript submitted for publication.

- The work was carried out at the University of Zürich; the funding information should have also acknowledged funding by Kanton Zürich.

- Work carried out by Noll’s group in order to test the data reported in Tables 1 and 2 showed rescue efficiencies of survival and male fertility considerably lower than those reported in the article. The authors have indicated that they do not have access to the raw data on which the results are based, it is therefore currently not possible to verify the results or to carry out an adequate comparison of the data against those obtained by Noll’s group.

The authors would like to apologise to the readers and Dr Noll.

